# Intimate partner violence help-seeking norms: scale reliability and cross-sectional multilevel associations with intimate partner violence among youth in Nairobi, Kenya

**DOI:** 10.1136/bmjopen-2023-080699

**Published:** 2025-01-14

**Authors:** Anaise Williams, Shannon N. Wood, Mary Thiongo, Peter Gichangi, Bianca Devoto, Kristin G. Bevilacqua, G Wamue-Ngare, Michele R. Decker

**Affiliations:** 1 Department of Population, Family and Reproductive Health, Johns Hopkins University Bloomberg School of Public Health, Baltimore, Maryland, USA; 2 International Centre for Reproductive Health Kenya, Mombasa, Kenya; 3 Technical University of Mombasa, Mombasa, Kenya; 4 Department of Sociology, Gender and Development Studies, Kenyatta University, Nairobi, Kenya; 5 Women’s Economic Empowerment Hub, Kenyatta University, Nairobi, Kenya

**Keywords:** Health Equity, Adolescent, PUBLIC HEALTH

## Abstract

**Abstract:**

**Objectives:**

Intimate partner violence (IPV) threatens women’s health and safety. Support services can mitigate the impact, yet few survivors seek services in part due to social norms that discourage use. Little agreement exists on how to measure norms and attitudes related to IPV help-seeking. The objectives were to (1) refine an IPV Help-Seeking Attitudes Scale and examine its psychometric properties, (2) explore differences in attitudes between young men and young women, and (3) examine associations of past 6-month IPV among young women with the scale at the individual level (individual attitudes) and the by-gender community-aggregated averages (community norms) among youth in Nairobi, Kenya.

**Design:**

This cross-sectional, secondary analysis used data from a phone-based survey with a cohort of young men and young women recruited via respondent-driven sampling from April to May 2021. Cross-sectional exploratory factor analysis assessed underlying latent constructs. Multilevel mixed-effects models assessed associations with IPV experience for young women.

**Setting and participants:**

A youth cohort of 586 men and 591 women aged 15–24 years in Nairobi, Kenya.

**Primary outcome measure:**

Past 6-month physical and/or sexual IPV among young women.

**Results:**

The IPV Help-Seeking Attitudes Scale had acceptable internal reliability (Cronbach’s alpha >0.60). IPV prevalence was 17.5%, among whom 21.7% had sought any help for the past 6-month IPV. A one-unit increase in the community aggregate IPV Help-Seeking Attitudes Scale among young women was associated with reduced odds of IPV (aOR: 0.17, 95% CI: 0.03–0.98). However, the individual-level attitudes scale was not associated with IPV nor was the men’s community aggregate scale.

**Conclusions:**

The IPV Help-Seeking Attitudes Scale had adequate psychometric properties. The results suggest that young women living in a community where the norm among women enables IPV response may have a reduced risk of IPV. Community norms change to better enable IPV response among young people may help reduce IPV and increase help-seeking.

**STRENGTHS AND LIMITATIONS OF THIS STUDY**

- Reliable measures of norms surrounding intimate partner violence (IPV) help-seeking are limited, particularly among youth. Our study demonstrates the reliability of a scale inclusive of a subset of items from an existing scale.
- Our study applies a multilevel approach to examine the influence of IPV help-seeking attitudes at both individual and community levels on IPV among young women in Nairobi, which extends the field past a historical focus on individual-level influences.
- The secondary analysis approach limits the study to scale refinement rather than a full-scale development study.
- Our IPV-experienced sample size is small, and help-seeking was quite limited, which limited statistical power to examine normative influences on help-seeking experiences.

## Background

 Gender equality is a key component of the Sustainable Development Goals, reflected in Goal 5.^1^ Yet nearly one in three women globally experience intimate partner violence (IPV), that is, physical, sexual, or emotional violence perpetrated by an intimate partner.^2^ IPV onset often occurs in adolescence,^3^ and experiencing IPV early in life increases the risk of adulthood victimisation.^4 5^ Adolescent girls and young women are at high risk for IPV: data across 15 low- and middle-income countries suggest that past-year physical IPV among girls ages 13–19 is as high as 23%.^6^ IPV among adolescent girls and young women may be even higher in Kenya, where an estimated 25% of those ages 13–19 years report past 12-month physical IPV.^7^ As such, the Kenyan government has highlighted the need to expand survivor support services and has included this as a key action item within Kenya’s roadmap for advancing gender equality.^8^

Integrating gender-based violence prevention and response across sectors is one of the three pillars of comprehensive violence prevention and response,^9^ along with a focus on at-risk populations such as girls and young women. Safe spaces for survivors to disclose abuse and obtain psychosocial or medical support are a key part of the response;^10^,^12^ it can reduce post-traumatic stress,^12^ self-blame^13^ and revictimisation.^14^,^16^ Yet, globally, most women who experience IPV do not seek help.^17^ Low rates of help-seeking were first established in the WHO multicountry study in 2005, with little evidence of meaningful change since then.^18^ Women seek informal help from friends or family more often than they seek formal help from legal or health systems due to trust, comfort and accessibility.^19^ Norms are understood to be a key contextual factor affecting IPV experience and help-seeking, as characterised by Heise’s ecological model of gender-based violence.^20^ Social norms supporting IPV tolerance and gender inequity in economic and social spaces are believed to discourage women from seeking both formal and informal support.^21^,^24^ Youth, in particular, may be less able to navigate informal and formal support systems for help when IPV occurs, while simultaneously being more influenced by the normative expectations of their peers.

While personal attitudes are internally motivated judgements about behaviour, social norms are the perceived or actual beliefs of the people in one’s social network or community.^25^ Evidence exists that social norms may have more impact than individual-level attitudes on IPV experiences and help-seeking. For instance, a qualitative study in Tanzania found that women who individually believed that IPV should be acted on were often blocked from formal and informal help-seeking due to prevailing societal norms.^19^ A qualitative study in Rwanda found that even if women had personal attitudes that supported leaving abusive relationships, the social norms in their community around family and marriage often prevented IPV response.^26^ While the role of norms in women’s help-seeking is recognised, a knowledge gap persists in effectively measuring specific attitudes and norms related to IPV help-seeking in low- and middle-income settings.^2427^,^30^ One scale capturing help-seeking attitudes is the IPV Help-Seeking Norms Scale (IPV-Help), which was tested in Nepal among married women, though it has not been fielded in other settings, including Kenya.^31^ The scale performed relatively well, with author recommendations including testing among men and youth. The IPV-Help scale has item overlap with the Partner Violence Norms Scale (PVNS), a scale also tested in Nepal with strong confirmatory factor analysis fit statistics (RMSEA=0.07) and Cronbach’s alpha (0.85).^32^ While several scales exist that capture attitudes towards IPV help-seeking, few have been tested among both young men and women in low- and middle-income contexts.^32^,^37^ Few studies go beyond attitudes to study norms, examining individual-level attitudes without considering aggregate community metrics.^38^ While several data sources measure IPV justification among women and men, namely, the Demographic and Health Surveys, few explore individual attitudes towards IPV help-seeking. Even fewer studies survey men on factors influencing women’s IPV help-seeking. This gap limits a fuller understanding of the social norms landscape around community responses to IPV cases, in which men play a critical role.

Given the need for research on attitudes influencing IPV help-seeking, this secondary analysis among young men and young women in Nairobi, Kenya, aimed to (1) examine the psychometric properties of a scale (IPV Help-Seeking Attitudes) generated from existing items to capture attitudes towards IPV justification, community intervention, and informal and formal service help-seeking; (2) explore differences between young men and women; and (3) examine associations of past 6-month IPV among young women with the scale at the individual level (individual attitudes) and the by-gender community aggregated averages (community norms). In exploring by-gender community norms, we explore the question of whether there are differences in the effects of men vs women peers’ attitudes on IPV experience among young women. To enhance relevance and extend past research, items were drawn from the IPV-Help Scale and refined based on local priorities; the full set of tested items included three items from the World Values Survey^39^ on tolerance of gendered economic disparities to enhance local relevance and context.

## Methods

### Study sample

The data for this study’s secondary data analysis drew from a subsample of youth participants within the larger Nairobi Youth Respondent Driven Sampling Survey, an ongoing cohort study of adolescents and young adults. Eligible participants for the original cohort in 2019 were aged 15–24 years and residing in Nairobi for at least one year. In 2019, all respondents were unmarried, but 61% reported being involved in a romantic relationship. Participants were recontacted in 2020 and subsequently six months later in 2021, where 87% (1177/1317) of the original sample verbally consented and was surveyed. In this round, help-seeking attitude questions were integrated for the first time in response to local needs. The 2021 sample of young men (n=586) and young women (n=591), ages 16–26, was the analytic sample for the present secondary analysis. Additional sampling and recruitment details can be found elsewhere.^40^

Trained resident enumerators (REs) collected data in English or Swahili using OpenDataKit (ODK) on tablets or smartphones. Data collection was conducted by phone. Questions on IPV were asked only among partnered young women. REs were trained in sensitive data collection and received specialised training specific to gender-based violence (GBV) protections, and all data were collected following best practices for GBV research.^41^ REs asked participants about their safety and privacy before beginning data collection. REs gave participants a ‘safe phrase’ to discreetly report a privacy breach during data collection. If the safe phrase was used, participants were given the option to reschedule. Participants were instructed that they could skip any question they did not wish to answer. GBV support services were provided within a larger list of supports to minimise risk. Participants received 500 KES or US$5 per survey completed.

### Measures

#### Survey items considered for scale refinement

Seven items were explored: (1) husbands may use force to reprimand their wives because men should be in control of their families (while focused on tolerance of IPV rather than direct help-seeking, this item is included by the original scale authors because the attitude measure is an important precursor to seeking help; when we drop the item “husbands may use force to reprimand their wives because men should be in control,” the alpha drops to 0.67 from 0.71, suggesting that this item increases internal consistency); (2) a woman who complains about her husband’s violent behaviour is considered disloyal; (3) a woman who seeks help from police for domestic violence brings shame on her family; (4) women’s groups who get involved in situations of domestic violence usually make the situation worse; (5) when jobs are scarce, men should have more right to a job than women; (6) if a woman earns more money than a boyfriend or husband, it can cause problems; and (7) having a job is the best way for a woman to be an independent person. Respondents indicated their level of agreement on a 5-point Likert scale (strongly agree, mostly agree, neutral, mostly disagree or strongly disagree). Items (1)–(4) were drawn from the IPV-Help Scale and items (5)–(7) from the World Values Survey (online supplemental annex table 1). Economic agency and participation among women are known to be a key factor in help-seeking; for instance, working women have been found to be more likely to seek help for IPV.^42^ Though items (5)–(7) do not explicitly target help-seeking, we were interested in exploring attitudes towards broader economic gender equity in alignment with help-seeking attitudes.

**Table 1:** Sample characteristics, weighted

	All % (n=1177)	Men % (n=586)	Women % (n=591)
Education completed*			
Primary or below	32.9	31.8	33.6
Above primary	67.1	68.2	66.4
Age			
16–19 years	22.7	20.2	24.4
20–26 years	77.3	79.8	75.6
Main activity			
Student/caregiver/other	49.9	39.9	56.9
Paid work	50.1	60.1	43.1
Basic financial needs			
Able to meet needs	54.4	61.9	49.1
Not able to meet needs	45.6	38.1	50.9
Partnered, within the past 6 months			
Non-partnered	35.2	33.5	36.4
Partnered	64.8	66.5	63.6
IPV† past 6 months, among ever-partnered women (n=404)			
No	--	--	82.5
Yes	--	--	17.5
Help-seeking, among women who experienced IPV (n=67)			
No	--	--	78.3
Yes	--	--	21.7

* Measured in 2020 (6 months prior to 2021 survey).

† Inclusive of sexual and physical IPV past 6 months among partnered women.

#### IPV

IPV measures used behavioural assessment per best practices.^41^ Specifically, the survey asked women participants only: ‘In the past 6 months, has a partner ever pushed you, thrown something at you that could hurt you, punched or slapped you?’ and ‘In the past 6 months, have you had sex with a partner when you did not want to due to threats, pressure or force?’ The binary measure of IPV was coded as ‘1’ if the respondent answered ‘once’, ‘a few times’ or ‘often’ and ‘0’ if she responded ‘never’ to either of the mentioned IPV survey items.

#### Help-seeking

Women who reported any IPV were asked: ‘Did you seek help for any experiences of harm or unwanted sex?’ The binary measure of help-seeking was coded as ‘1’ if the respondent answered ‘yes’ and ‘0’ if the respondent answered ‘no’.

#### Covariates

Measures included in multivariate models were current job status (work for pay vs caregiver or student), age (below vs above 19 for bivariate analysis, continuous for multivariate regression modelling), school completed (above vs below secondary) and ability to meet basic needs. The ability to meet basic needs was assessed via a 4-point Likert scale (very able, somewhat able, not very able and not at all able) and dichotomised for analysis as very able/somewhat able vs not very able/not at all able.

#### Subcounty aggregate norms

To capture community norms rather than individual attitudes, an aggregate scale score by Nairobi subcounty (n=18) was created through taking the weighted mean score for young men and women separately. While the scale at the individual level is a proxy for individual attitudes, the subcounty aggregate scale is a proxy of the gender-specific community norm.

### Statistical analysis

#### Exploration of survey items

Sample characteristics were explored overall and by gender. Among young women only, IPV and help-seeking prevalence were reported. The percent agreement with each of the seven items under consideration for a scale was presented for both genders. Adjusting for weighting and survey design, agreement with each item and significance across genders was assessed.

#### Scale refinement

Exploratory factor analysis of the seven survey items considered for scale refinement employed a polychoric correlation matrix with promax rotation to examine evidence of an underlying latent construct, both overall and by gender. For the items that fell together based on eigenvalue (>1) and factor loadings (>0.40), a scale was constructed.^43^ Analysis of scale reliability focused on interitem reliability, using both Cronbach’s Alpha Coefficient and the Omega Coefficient.

#### Scale associations

Scale average scores by covariates listed above, overall and by gender, and by IPV and help-seeking among partnered young women, were explored. Significance testing of whether the scale varied by covariate, stratified by gender, was assessed using linear regression. The distribution of individual-level scale scores within and across subcounties overall and by gender was explored using box plots. Given the clustering of individual responses to scale survey items within subcounties, multilevel modelling was used to correct standard errors to accurately estimate subcounty/community influences on the outcome of interest.^44^ Mixed-effects multilevel logistic regression with random intercepts for subcounty associated individual-level IPV with individual-level attitudes and gender-specific subcounty aggregate norms measures, adjusting for age, education, whether the woman is working, ability to meet basic financial needs and recruitment clustering. Adjusted ORs (aORs) were presented between the scale score and IPV at the individual level (comparing differences in the outcome between two people with differing individual attitudes who live in the same subcounty) and at the subcounty level (comparing differences in the outcome between two people with the same individual attitudes but living in subcounties with differing aggregate norms scores), for IPV experience among young, partnered women. IPV was not measured among young men. Given the small sample size of young women who experienced IPV, we were unable to associate the scale with help-seeking itself.

All analyses were conducted using Stata 17.0 (College Station, TX) with statistical analysis set a priori at p<0.05. The analytic sample was restricted to observations with no missing values for the seven attitude measures. No missing values were present across independent or covariate variables within the analytic sample. Sampling weights accommodate the RDS study design using RDS-II (Volz-Heckathorn) weights, postestimation adjustment based on 2014 KDHS population data (age, sex and education levels) and modest adjustment for loss-to-follow-up. All presented estimates are weighted unless otherwise noted, and statistical testing accounts for clustering among participants recruited by the same recruiter at baseline.

### Patient and public involvement

The data used for this analysis were drawn from a broader community-engaged Kenya-based study, which engaged public and youth input at all phases. During the formative research stage before the 2019 cohort recruitment, input from community-based, youth-serving organisations informed the study recruitment strategy for feasibility, survey measures, and constructs to ensure local relevance and study logistics to maximise participant comfort and confidentiality. All recruitment and procedures were conducted by trained resident enumerators selected from underlying communities and who provided inputs on measures for clarity and aided in result interpretation. The ongoing cohort data collection aims to collect measures on violence risk, help-seeking, and norms among youth in Nairobi that are grounded in local needs. Measures on help-seeking attitudes were added for the 2021 survey round in response to in-country priorities to extend what is available in other publicly available datasets, such as the Kenya Demographic and Health Surveys.

## Results

### Exploration of survey items

Over half of the sample, 67.1% had above primary education, with similar percentages between young men and young women (table 1). About 77% of the sample was 20–26 years old, and all participants were older than 15 years at the time of the survey. Approximately half of the sample’s main activity was paid work, whereas the other half was school, caregiving or something else. Young men were more likely to report paid work as their main activity than young women (60.1% vs 43.1%). Slightly less than half, 45.6%, reported being unable to meet basic financial needs. Young women were more likely to report being unable to meet basic needs, 50.9%, compared with men, 38.1%. Over half of the sample, 64.8%, reported having an intimate partner within the past six months. Among young women who reported having an intimate partner in the past six months (n=404), 17.5% reported any sexual or physical IPV within the past six months. Among young women who reported any IPV (n=67), 21.7% reported seeking help from either formal or informal sources.

Young women consistently had more enabling IPV help-seeking attitudes than young men (online supplemental annex table 2), and four of seven survey items significantly differed by gender. Specifically, young men were significantly more likely to agree with the statements: ‘husbands may use force to reprimand their wives because men should be in control of their families’ (p<0.001), ‘women’s groups who get involved in situations of domestic violence usually make the situation worse’ (p<0.001) and ‘when jobs are scarce, men should have more right to a job than women’ (p<0.001). Young women were significantly more likely than young men to agree with the statement ‘having a job is the best way for a woman to be an independent person’ (p<0.001).

**Table 2:** Factor loading results from factor analysis, overall and by gender

	Overall	Young men	Young women
Husbands may use force to reprimand their wives because men should be in control of their families	0.64	0.67	0.59
A woman who complains about her husband’s violent behaviour is considered disloyal	0.74	0.71	0.77
A woman who seeks help from the police for domestic violence brings shame on her family	0.74	0.75	0.73
Women’s groups that get involved in situations of domestic violence usually make the situation worse	0.61	0.65	0.55
Eigenvalue	1.86	1.94	1.77
Cronbach’s alpha	0.71	0.74	0.67
Omega coefficient	0.72	0.74	0.68
Observations	1177	586	591

### Scale refinement

Exploratory factor analysis of the seven items with promax rotation suggested two underlying factors with eigenvalues 2.46 and 1.70, grouping items 1–4 on Factor 1 and items 5–7 on Factor 2. For items 5–7 (Factor 2), there were small factor loadings of 0.34, 0.50 and 0.12 and a low three-item Cronbach’s alpha of 0.32. These three items therefore did not suggest strong enough inter-reliability to justify a separate subscale. Running items 1–4 together in factor analysis demonstrated factor loadings greater than 0.60 and a Cronbach’s alpha of 0.71 (table 2). Therefore, only items 1–4 were included in the final IPV Help-Seeking Attitudes Scale. Young men typically displayed stronger factor loadings than young women, and Cronbach’s alpha for young men was higher than for young women (0.74 vs 0.67). Checking internal reliability using the Omega coefficient generated a similar output to Cronbach’s alpha. Higher values of the IPV Help-Seeking Attitudes Scale signified more egalitarian attitudes. Among the full sample, the IPV Help-Seeking Attitudes Scale ranged from 4 to 20 with a mean of 15.0 (SD=3.0). The scale was lower among young men (mean=14.3) compared with young women (mean=15.5) (table 3).

**Table 3:** IPV Help-Seeking Attitudes Scale by sample characteristics, weighted (n=1177; 586 men; 591 women)

	IPV Help-Seeking Attitudes Scale*			Alpha		
	All	M	W	All	M	W
Overall						
	15.0	14.3	15.5	0.71	0.74	0.67
Education						
Primary or below	15.0	14.3	15.5	0.72	0.78	0.61
Above primary	15.0	14.4	15.4	0.71	0.73	0.69
P value†	0.803	0.847	0.655	*--*	*--*	--
Age						
16–19 years	15.2	14.3	15.7	0.73	0.77	0.63
20–26 years	14.9	14.3	15.4	0.71	0.73	0.67
P value†	0.363	0.978	0.368	*--*	*--*	--
Main activity						
Student/caregiver/other	15.0	13.8	15.5	0.71	0.75	0.66
Paid work	15.0	14.7	15.4	0.71	0.73	0.68
P value†	0.797	*0.045*	0.556	*--*	--	--
Basic financial needs						
Able to meet needs	15.0	14.1	15.8	0.73	0.73	0.71
Not able to meet needs	15.0	14.6	15.2	0.69	0.75	0.61
P value†	0.871	0.229	*0.024*	*--*	*--*	--
Partnered						
Non-partnered	15.2	14.4	15.8	0.76	0.80	0.68
Partnered	14.9	14.3	15.3	0.69	0.71	0.66
P value†	0.179	0.938	0.078	*--*	*--*	--
Physical or sexual IPV past 6 months, among ever-partnered women						
No	--	--	15.5	--	--	0.66
Yes	--	--	14.1	--	--	0.61
P value†	*--*	*--*	*0.003*	*--*	*--*	*--*
Help-seeking, among women who experienced IPV						
No	--	--	14.4	--	--	0.68
Yes	--	--	13.3	--	--	0.59
P value†	*--*	*--*	*0.254*	*--*	*--*	*--*

Italics indicate p<0.05

* Sum of four items (higher is more egalitarian); significantly differs by gender (pp<0.001).

† P- value of linear regression of demographic variable on score adjusting for weighting and survey sampling design.

### Scale associations

Overall and among young men and women separately, there was no significant difference in the IPV Help-Seeking Attitudes Scale between participants with below and above primary education (table 3). Among men, the scale was significantly more egalitarian among those whose main activity was paid work (p=0.045). Women who were not able to meet basic needs were significantly lower on the scale as compared with women who were able to meet basic needs (15.8 vs 15.2, p=0.024). Among women, the scale slightly differed by partnership status, with partnered women having lower scores (p<0.10). Alpha levels were comparable by whether basic needs were met for men, though internal consistency was higher among women who had their basic needs met as compared with women who did not (0.71 vs 0.61). The alpha scores were lower among partnered young people (0.69) as compared with nonpartnered young people (0.76).

For the subsample of partnered young women (n=404), those who had experienced IPV were significantly lower on the IPV Help-Seeking Attitudes Scale than those who had not experienced IPV (p<0.01) (table 3). The scale’s internal consistency was lower among partnered young women who had experienced IPV than partnered women who had not experienced IPV (alpha levels of 0.61 vs 0.66). For the subsample who had experienced IPV (n=67), there was not a significant difference in the scale score between those who sought and did not seek help. The alpha level for the scale was lower among those who did seek help (0.59 vs 0.68).

Differences in variation of the IPV Help-Seeking Attitudes Scale across subcounties signified some evidence of subcounty clustering and the need to adjust for clustering in inference modelling (online supplemental annex figure 1). Table 4 presents results from mixed-effects logistic regression. In adjusted analyses, no significant association between the IPV Help-Seeking Attitudes Scale at the individual level and IPV experience was identified (aOR: 0.88; 95% CI: (0.75–1.04)). A young woman who lived in a subcounty with a one-unit increased aggregate IPV Help-Seeking Attitudes Scale among young women had significantly reduced odds of experiencing IPV (aOR: 0.17; 95% CI: (0.03, 0.98)), compared with a young woman of the same earner status, age, education completion, and ability to meet basic needs but living in a subcounty with a one-unit lower norms score among women. No significant association was identified between the aggregate IPV Help-Seeking Attitudes Scale among young men and women’s IPV experiences.

**Table 4:** Mixed-effects logistic regression of individual attitudes and aggregate norms on women’s past 6-month physical or sexual IPV experiences, weighted

	Physical or sexual IPV in the past 6 monthsaOR (95% CI)
Individual level	
Women’s IPV response attitudes†	0.88 (0.75–1.04)
Subcounty level	
Men’s aggregate IPV response norms†	1.72 (0.88–3.35)
Women’s aggregate IPV response norms†	0.17* (0.03–0.98)
Observations	404

Models account for survey weighting and clustering at the subcounty level, adjust for age, education, whether the woman is working, ability to meet basic financial needs, and recruitment clustering.

*p<0.05 protective

†Higher = more protective.

aOR, adjusted OR

## Discussion

Reliable measures of attitudes and norms that affect help-seeking are limited. This study explores a shortened version of the IPV-Help scale, previously tested among married women in Nepal^31^ and refined for an urban youth population in Nairobi, Kenya. The refined scale (IPV Help-Seeking Attitudes Scale) demonstrated strong performance among urban youth, affording valuable insight into measurement performance and youth-held attitudes towards IPV help-seeking. Findings suggest that community-level IPV help-seeking norms (attitudes aggregated at the subcounty level) may be more strongly associated with IPV experience than individual attitudes, highlighting a link between the community help-seeking normative context and IPV experience. Specifically, while individual help-seeking attitudes did not correlate with IPV, women living in subcounties with more protective women’s *community* norms for IPV help-seeking were less likely to report IPV experience. Interestingly, the community norms on IPV help-seeking among young men were not correlated with young women’s IPV experiences. With increased recognition of IPV among youth and a resurgence of interest in access to support services comes a critical need to monitor norms that influence help-seeking, the IPV Help-Seeking Attitudes Scale may be a valuable first step to monitoring community norms that may enable or inhibit access to those services.

While adequately reliable overall, the IPV Help-Seeking Attitudes Scale was less internally consistent among young women (0.67) than young men (0.74) and even less reliable among partnered young women who had experienced IPV (0.61). The limited reliability for young women who have experienced IPV means the IPV Help-Seeking Attitudes Scale was less strong in capturing individual-level attitudes towards IPV help-seeking among women who experienced IPV. This lower internal reliability may reflect limited statistical power due to this smaller subsample; there may also be high variability in attitudes towards help-seeking within this population, potentially due to varied lived experiences with prior help-seeking. The results highlight the importance of measuring attitudes and norms among young women, particularly those with lived experiences of violence, to better understand and promote IPV help-seeking. Further research is needed to continue to improve measures in this area, particularly among young women who have experienced partner violence.

Attitudes and social norms around help-seeking are one component of a myriad of barriers to seeking help, inclusive of availability and accessibility to formal services, quality of services, legal landscapes, and financial factors. Less than a quarter of the young women sought help for IPV experience, despite the relatively strong availability of services in Nairobi, including the Gender Violence Recovery Centre^45^ and trained healthcare providers.^46^ The Kenyan Government’s 2021 GBV Roadmap calls for a multisectoral approach to GBV response, including coverage of GBV response in the essential minimum package of the Universal Health Coverage by 2022.^8^ Further, the global GBV strategy calls for supportive environments for help-seeking^9^; this cannot be achieved through infrastructure only, rather, community mobilising is needed to create a normative environment that facilitates, rather than impedes, accessing IPV support services. The finding that the men’s collective community norm did not correlate with IPV experience calls for more research on how to involve young men in IPV cessation efforts.

The study has several additional limitations. Foremost, this study was a secondary data analysis using data collected with an ongoing cohort of youth; the larger study was not specifically focused on IPV help-seeking attitudes nor on scale development surrounding this issue. Individual survey measures did not address broader structural factors that impact help-seeking, such as gender-equitable cash control and decision-making. Individual items for the scale drew from existing, validated scales and were chosen based on local priorities with input from Kenya-based partners working with youth in Nairobi. The study did not include qualitative formative scale development elements nor was a fuller set of items included, both of which would have been informative for refining items specific to a young population and the Kenyan context. The results provide a foundation for a more detailed scale development study on this topic in which rigorous criterion validity, construct validity, and test–retest reliability testing can be applied. Cross-sectional, retrospective data limit causal inference. This is particularly challenging in examining attitudes among those with IPV experience. Surveys were conducted via mobile phone, which may have caused response bias and threatened external validity; however, postestimation weights improve generalisability to the youth population in Nairobi. The IPV-experienced sample size is small, which limited statistical power to explore associations between the scale and IPV and was prohibitive for examining help-seeking as a critical outcome. Future research with larger sample sizes and statistical power should explore this further, as well as the protective trend linking enabling norms among women and IPV experiences. Additional measurement limitations include those related to the nature, quality and sources of help-seeking; moreover, the IPV measure is limited to sexual and/or physical IPV only and does not inform on other forms of IPV that are common among youth and can vary across the life course, such as tech-facilitated abuse, controlling behaviour, economic abuse, and psychological abuse.

The results are actionable for practitioners and policy makers. Recommendations include addressing not only individual attitudes but also community-level norms on violence and help-seeking. Changing norms to enable, rather than impede, the uptake of IPV supports remains an important goal to reduce barriers to care. Several IPV prevention programmes that have addressed community norms can be considered models. Specifically, the SASA! Initiative in Uganda uses community mobilisation to change gender equality norms that affect IPV and strengthen the community response to IPV, with significant reductions in both IPV acceptance among women and reductions in past-year physical IPV.^47^ In Ghana, the Gender Centre’s Rural Response Strategy used community-based action teams to challenge community members’ attitudes and support referral to social services for IPV help-seeking and significantly reduced sexual IPV among women after 18 months.^48^ Current study findings add to the body of literature affirming the importance of changing collective attitudes for IPV prevention and response. The high prevalence of IPV among young women, and the normative climate that risks undermining help-seeking, demands action to ensure the health, safety and well-being of young women and their communities in urban Kenya.

## supplementary material

10.1136/bmjopen-2023-080699 online supplemental file 1

10.1136/bmjopen-2023-080699 online supplemental file 2

10.1136/bmjopen-2023-080699 online supplemental file 3

## Data Availability

Data are available upon reasonable request from pmadata.org.
