# Supplementary figures and images for: Intimate partner violence help-seeking norms: scale reliability and cross-sectional multilevel associations with intimate partner violence among youth in Nairobi, Kenya
Source: BMJ Open. 2025 Jan 14;15(1):e080699. doi: 10.1136/bmjopen-2023-080699 (PMC11751895; doi:10.1136/bmjopen-2023-080699)

Annex Figure 1: Distribution of IPV Help-seeking Attitudes Scale within and across subcounties

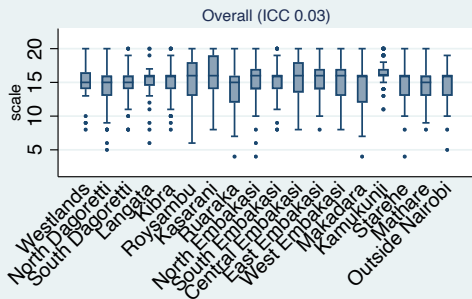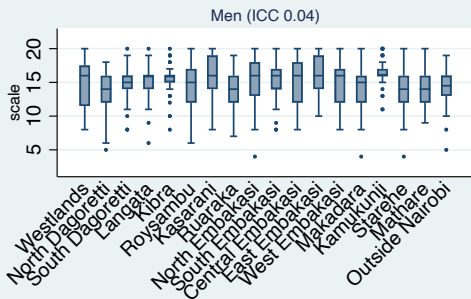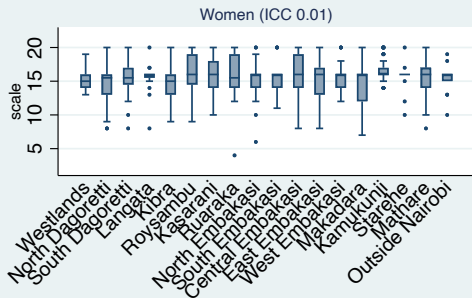

Supplement: online supplemental file 1 [file bmjopen-15-1-s001.pdf]
