# Supplementary material for: Intimate partner violence help-seeking norms: scale reliability and cross-sectional multilevel associations with intimate partner violence among youth in Nairobi, Kenya
Source: BMJ Open. 2025 Jan 14;15(1):e080699. doi: 10.1136/bmjopen-2023-080699 (PMC11751895; doi:10.1136/bmjopen-2023-080699)
Supplement: online supplemental file 2 [file bmjopen-15-1-s002.pdf]

Supplemental Material

Annex Table 1: Survey item sources

IPV help-seeking norms scale (IPV-Help)[1]	World Values Survey - Right to Employment[2]	Partner Violence Norms Scale (PVNS)[3]	Current Scale Analysis
		A husband who helps his wife with the household chores will not be respected by his family	
		A man who makes important decisions jointly with his wife will be considered a weak man by his family	
		A man's family will think he is a disloyal son if he takes his wife's opinion over his mother's opinion	
		A woman who openly expresses her sexual desires to her husband is perceived to be vulgar	
Husbands may use force to reprimand their wives because men should be in control of their families		Husbands may use force to reprimand their wives because men should be in control of their families	Included
A woman who complains about her husband’s violent behavior is considered a disloyal wife by her in-laws		A woman who complains about her husband's violent behavior is considered a disloyal wife by her in-laws	Included
A woman who does not tolerate violence from her husband is dishonoring her family and should not be welcomed home		A woman who does not tolerate violence from her husband is dishonoring her family and should not be welcomed home	
A woman who seeks help from the police for domestic violence brings shame on her family and should not be welcomed home			Included
A person who intervenes when a woman is being beaten by her husband would be considered to be interfering in the couple’s private affairs.		A person who intervenes when a woman is being beaten by her husband would be considered to be interfering or meddling in the couple's private affairs	
Mediation is the best solution for families who experience domestic violence			
A woman should tolerate violence to keep her family together			
Women’s groups who get involved in a case of domestic violence usually make the situation worse.			Included
Men should seek the advice of community leaders before allowing a female family member to seek help from a security and justice provider			
	When jobs are scarce, men should have more right to a job than women.		Did not fit with other items
	When jobs are scarce, employers should give priority to people of this country over immigrants.		
	If a woman earns more money than her husband, it’s almost certain to cause problems.		Did not fit with other items
	Having a job is the best way for a woman to be an independent person.		Did not fit with other items

[1]Clark, C. Development and Measurement Properties of the IPV Help-Seeking Norms Scale. Unpublished presentation. 2017.
[2]Inglehart, R., C. Haerpfer, A. Moreno, C. Welzel, K. Kizilova, J. Diez-Medrano, M. Lagos, P. Norris, E. Ponarin & B. Puranen et al. (eds.). (2014). WVS 2010-2012 Wave, revised master, June 2012. https://www.worldvaluessurvey.org/WVSDocumentationWV6.jsp
[3]Clark, Cari Jo, et al. "Social norms and women's risk of intimate partner violence in Nepal." Social science & medicine (2018). 202; 162-169.
