# Supplementary material for: Intimate partner violence help-seeking norms: scale reliability and cross-sectional multilevel associations with intimate partner violence among youth in Nairobi, Kenya
Source: BMJ Open. 2025 Jan 14;15(1):e080699. doi: 10.1136/bmjopen-2023-080699 (PMC11751895; doi:10.1136/bmjopen-2023-080699)
Supplement: online supplemental file 3 [file bmjopen-15-1-s003.pdf]

Supplemental Material

Appendix Table 2: Survey items, (n=1,177; 586 men, 591 women), unweighted

	Strongly agree (1) row (%)		Mostly agree (2) row (%)		Neutral (3) row (%)		Mostly disagree (4) row (%)		Strongly disagree (5) Row (%)		Mean score		p-value difference between genders~
	W	M	W	M	W	M	W	M	W	M	W	M	
1.Husbands may use force to reprimand their wives because men should be in control of their families+	2.0	4.3	9.6	16.9	1.7	4.1	59.1	52.7	27.6	22.0	4.0	3.7	**<0.001**
2.A woman who complains about her husband's violent behavior is considered disloyal+	2.9	3.1	13.4	12.8	3.2	5.8	56.4	54.4	24.2	23.9	3.9	3.8	0.696
3.A woman who seeks help from police for domestic violence brings shame on her family+	1.4	3.1	10.0	12.6	5.3	4.3	58.7	54.8	24.7	25.3	4.0	3.9	0.115
4.Women's groups who get involved in situations of domestic violence usually make the situation worse+	2.7	4.4	14.4	22.9	10.5	11.1	57.2	46.8	15.2	14.9	3.7	3.4	**<0.001**
5.When jobs are scarce, men should have more right to a job than women*	2.0	3.8	8.0	13.8	5.4	9.2	59.7	52.9	24.9	20.3	4.0	3.7	**<0.001**
6.If a woman earns more money than a boyfriend or husband, it can cause problems*	8.3	10.4	35.5	34.1	8.0	9.6	36.9	32.8	11.3	13.1	3.1	3.0	0.646
7.Having a job is the best way for a woman to be an independent person*	46.4	25.4	46.9	55.3	1.7	5.8	4.2	11.1	0.9	2.4	1.7	2.1	**<0.001**

~p-value of t-test comparing survey item between genders
+From IPV-Help (Annex 1)
*From the World Values Survey (Annex 1); items (5-7) not included in further analysis
