# Supplementary material for: Intimate partner violence help-seeking norms: scale reliability and cross-sectional multilevel associations with intimate partner violence among youth in Nairobi, Kenya
Source: BMJ Open. 2025 Jan 14;15(1):e080699. doi: 10.1136/bmjopen-2023-080699 (PMC11751895; doi:10.1136/bmjopen-2023-080699)
Supplement: Supplementary file 4 [file bmjopen-2023-080699.reviewer_comments.pdf]

PEER REVIEW HISTORY

BMJ Open publishes all reviews undertaken for accepted manuscripts. Reviewers are asked to complete a checklist review form and are provided with free text boxes to elaborate on their assessment. These free text comments are reproduced below.

ARTICLE DETAILS

Title (Provisional)

Intimate partner violence help-seeking norms: scale reliability and cross-sectional multilevel associations with intimate partner violence among youth in Nairobi, Kenya

Authors

Williams, Anaise; Wood, Shannon; Thiongo, Mary; Gichangi, Peter; Devoto, Bianca; Bevilacqua, Kristin; Wamue-Ngare, G; Decker, Michele

---

VERSION 1 - REVIEW

**Reviewer**	**1**
**Name**	**Leight, Jessica**
**Affiliation**	**International Food Policy Research Institute**
**Date**	**25-Oct-2023**
**COI**	**I report no competing interests.**

This paper creates a new scale designed to capture social norms around using IPV response services and measures its psychometric properties, along with the associations between the new scale and IPV. The paper contributes to a very narrow literature around measuring norms linked to IPV response; there is one previous scale tested in Nepal among married women that is highly relevant, but few other examples.

This paper has the potential to be a useful contribution to the literature, as understanding norms around seeking services following IPV is important. I have a few suggestions for revisions.

1. First, the paper should be clearer about its contribution. The third paragraph of the introduction cites a number of scales and related papers in the literature, but several of these seem to be more general scales that capture norms around the acceptability of IPV. This seems to be conceptually distinct from norms around seeking services specifically after experiencing IPV; and if the goal is simply to have a measure of social norms around IPV, there are many candidates (and the contribution of this paper, specifically, is smaller). The authors should clarify that their goal is to capture norms around service utilization and similarly clarify what other papers, if any, have done so.

2. Closely related to this point, my biggest quibble with the exercise is the selection of

items. Items number 5 to 7 included in their scale seem prima facie unrelated to the other items and unrelated to the main topic of interest (responses to IPV); these items are not even explicitly about violence, much less about use of services following the experience of violence, and are more related to norms around women's economic role. The authors should explain why these items were selected and believed to be appropriate for the concept of interest.

3. Ultimately, of course, these items were excluded because they showed little coherence with the remaining items in factor analysis. This is unsurprising. The analysis should note that this is probably unsurprising; and I would also suggest that the authors note the inclusion of these largely unrelated items as a limitation. (They could have, instead, tested other items that more closely capture the concept of service-seeking after experience of IPV.)

4. I have a similar comment to make about item 1: husbands may use force to reprimand their wives because men should be in control. This is a statement about the appropriateness of IPV, not about seeking help following the experience of IPV. In this case, the item is retained based on its psychometric fit with other items, but this would also seem to merit more discussion. Why was this item selected for test- ing? Did the authors evaluate Cronbach's alpha when excluding this item? Note that the scale overall has an acceptable level of internal reliability, but arguably not an extremely high level.

5. Finally, it would be helpful to return again in the discussion to a comparison of this scale vis-a-vis the IPV-Help scale already evaluated in Nepal. The authors note that the scale in this paper is analyzed for youth, but is there a reason to believe this scale is particularly appropriate for youth, or particularly appropriate in Kenya? Do these scales have different or overlapping functions in the broader IPV measurement space?

**Reviewer**	**2**
**Name**	**Gardner , Jessica**
**Affiliation**	**Independent Researcher**
**Date**	**05-Jan-2024**
**COI**	**None**

The paper is well written and could provide valuable evidence to support response and prevention to intimate partner violence. The objectives, data sources, methods and results need more and clearer explanation for this work to make the contribution it seeks to.

Feedback on BMJ article
Intimate partner violence response norms: scale reliability and multilevel associations with intimate partner violence among youth in Nairobi, Kenya

1. The discussion on help-seeking is limited to the impact that individual attitudes and social norms have on behavior. Other barriers to help seeking from formal institutions, such as the availability, proximity, and accessibility of services, quality

and satisfaction with services provided, the legal framework such as mandatory reporting, and financial resources to enable help seeking, would be important to mention even if not the primary focus of the research.

2. The data sources for this analysis are unclear. It would help to say more about the primary data source(s), including an assessment of quality and validity for the purpose of this research. The section on Patient and Public Involvement could be subsumed into that as it appears to relate to the original data collection rather than this secondary analysis. Some commentary on the possibility to do this analysis to other existing data sources, such as the Demographic and Health Survey conducted in Kenya in 2014, which collected data on attitudes, experiences of IPV and help seeking.
3. Clearer explanation is needed on how the IPV Response Scale is constructed. In particular, how the 'IPV measure' and 'Help-seeking measure' are being used. 'IPV Response Scale' does not seem to be an appropriate title for something based only on attitudes to social norms. Especially as 'response' is such an established concept amongst practitioners working on IPV-related interventions.
4. Given the focus is on IPV related help-seeking, it could be more impactful to focus this analysis solely on the women respondents, or more clearly explain the value of including the men. With the focus on youth some mention of controlling behaviors and other forms of IPV that are routinely measured would help to highlight that forms of violence are often overlapping and may change during the life course, such as escalate during pregnancy.
5. The reason for, methods used and the findings from the subnational (subcounty) analysis could be explained in more detail.
6. Page 3, line 12-13 mentions that interpersonal violence is a leading cause of DALY among youth. Looking at the data source, this appears to be mainly firearm violence among males, and does not have any relationship with IPV. Including this statement could be misleading.
7. Page 3, line 26: the language "Identifying" survivors suggests a compromise of their privacy and confidentiality. The reference being quoted here appears to use language drawn from an outdated version of the 'United States strategy to prevent and respond to GBV globally'. I suggest quoting the primary source and updating the language to align with the 2022 version. Alternatively, consider referring to UN or other international guidance on the pillars of response and prevention.
8. Page 3, line 34: check reference to the year 2006 as is inconsistent with the publication date of reference 19.
9. Page 4, line 31: the objectives could be elaborated to explain why this work is being undertaken and how it will contribute to the field.
10. Page 4, line 54: In the description of the study sample, it would be important to elaborate on the partnership status being used in this study; the questions used to ascertain partnership status; and the percentage of the original and 2021 sample were ever-partnered (and therefore the focus on the study).
11. Page 5, line 48: provide reasons for why the last six months was used as a reference period as opposed to the standard last 12 months.
12. Page 8, line 26: consider using "intimate partner" in place of "romantic partner" for consistency.

13. Consider updating reference 2 to the 2018 WHO global estimates (Violence against women prevalence estimates, 2018: global, regional and national prevalence estimates for intimate partner violence against women and global and regional prevalence estimates for non-partner sexual violence against women. Geneva: World Health Organization; 2021. Licence: CC BY-NC-SA 3.0 IGO.)

**Reviewer**	**3**
**Name**	**Dabla, Vandana**
**Affiliation**	**USAID India**
**Date**	**05-Apr-2024**
**COI**	**None**

The study provides comprehensive background with clear objectives and methodology. Approach to data analysis as well as applied factorization is well defined. However, for the purpose of replicability of the study beyond specified geography of Nairobi, Kenya; inclusion of some additional points under methodology would strengthen the study e.g. process adopted to reduce the biases in case of self-reported data which is currently used in this study. In addition, description of recruitment or sampling of sub-group of YRDSS would be beneficial for easy replication of study. Overall, study objectives are relevant to the gender responsive system and it yields vital results.

**Reviewer**	**4**
**Name**	**Gonzalez-de la Torre, Hector**
**Affiliation**	**Universidad de Las Palmas de Gran Canaria, Enfermeria**
**Date**	**17-May-2024**
**COI**	**I do not have competing interests**

Dear Authors

Thank you for allowing me to review your manuscript. I am making some comments with the aim of improving this work.

-I have had difficulty reading your manuscript because I think there is a problem of structure. Given that you present two different objectives, I think it would be more coherent to present the study in phases or stages. In the first phase present the development of the scale and in a second phase the analysis of the associations, in this way the work would gain in clarity. The research seems to be well conducted, but it is presented in a disorganized manner. In my comments I will therefore make this distinction between a first phase, which would be the

development and evaluation of the properties of the scale, and in phase two, the analysis of the associations. Both phases should have a differentiated methodology and results.

Stage 1 Create an IPV Response Scale that captures attitudes towards IPV and IPV help-seeking and examine its psychometric properties

-The evaluation of psychometric properties of an instrument includes assessing content validation, construct, face validity, reliability (internal consistency and temporal stability, convergent-divergent validity among others. Indicate which psychometric properties have been finally examined and how.

-It seems that you have developed a scale from other existing scales. Indicate in a section which were the starting instruments and explain the process carried out: selected items, who made the selection of these and how the merging process was carried out. Cross-cultural and linguistic adaptation of the scales was made. In the end, do the selected items score differently? If this is the case, it has a decisive effect on the internal consistency and factor analysis.

-Explain and justify the estimated sample size.

-Indicate the approach used in the factor analysis (linear or nonlinear, Pearson's or polychoric correlation matrices) as well as the method of factor extraction. You can justify with references the system that you indicate of multiplying each survey item response by its loading based factorial load to create from there an adjusted scale.

-Why didn't you calculate the omega coefficient instead of the cronbach coefficient. It is better, especially when working with factor loadings.

VERSION 1 - AUTHOR RESPONSE

Thank you for the positive review of our paper, and to the reviewers for their incredibly thoughtful feedback. We are pleased to submit a revision that we feel has been greatly enhanced through the peer review process. We worked diligently to accommodate the reviewer requests for clarifications in the methodology and focus. We have updated the title and stated the consent process for participants. Please note we also in this process identified a small error in our multilevel regression in which we should have adjusted for clustering; some edits to original output for the associational table have been made, with no changes to the findings. Of note, in seeking a more streamlined paper, we realized that the adjusted scale was adding limited value and there are divergent views in the field as to how valuable this is; to save space we have removed this analysis. We hope you will agree that this enhances the paper and is responsive to reviewer inputs. A detailed response follows. Thank you for your continued consideration.

Reviewer #1: This paper creates a new scale designed to capture social norms around using IPV response services and measures its psychometric properties, along with the associations between the new scale and IPV. The paper contributes to a very narrow literature around measuring norms linked to IPV response; there is one previous scale tested in Nepal among married women that is highly relevant, but few other

examples. This paper has the potential to be a useful contribution to the literature, as understanding norms around seeking services following IPV is important. I have a few suggestions for revisions.

1.1 **First, the paper should be clearer about its contribution. The third paragraph of the introduction cites a number of scales and related papers in the literature, but several of these seem to be more general scales that capture norms around the acceptability of IPV. This seems to be conceptually distinct from norms around seeking services specifically after experiencing IPV; and if the goal is simply to have a measure of social norms around IPV, there are many candidates (and the contribution of this paper, specifically, is smaller). The authors should clarify that their goal is to capture norms around service utilization and similarly clarify what other papers, if any, have done so.**

Thank you for this comment. We consider one of our main contributions to be the exploration of these items 1) among youth, 2) among men in addition to women, and 3) both in terms of individual attitudes and as aggregate norms effects. We have adjusted our aims section of the paper, and hope this makes the unique contributions of the paper clearer (lines in clean manuscript; 139-150).

1.2 **Closely related to this point, my biggest quibble with the exercise is the selection of items. Items number 5 to 7 included in their scale seem prima facie unrelated to the other items and unrelated to the main topic of interest (responses to IPV); these items are not even explicitly about violence, much less about use of services following the experience of violence, and are more related to norms around women's economic role. The authors should explain why these items were selected and believed to be appropriate for the concept of interest.**

We acknowledge these items are much broader and speak to the economic gender equity landscape rather than IPV response norms directly, and indeed we did not expect them to fall in one single scale, as we found. We wanted to include these items in initial analysis for contextual understanding and to explore whether these items fell into their own scale. However, since they did not fit with the first four items nor held well as their own scale, they were dropped from further analysis. Under methods, we have added more context and justification for why we initially included these items (187-190).

1.3 **Ultimately, of course, these items were excluded because they showed little coherence with the remaining items in factor analysis. This is unsurprising. The analysis should note that this is probably unsurprising; and I would also suggest that the authors note the inclusion of these largely unrelated items as a limitation. (They could have, instead, tested other items that more closely capture the concept of service-seeking after experience of IPV.)**

We agree it is important to highlight our limited measures and clarify that this was secondary data analysis rather than a full scale development study. We were interested in the broader economic gender equity context and its influence on help-seeking but were limited by the measures available to us in this analysis. We have included significant additions to the limitations section to clarify these measurement limitations (383-393).

1.4 **I have a similar comment to make about item 1: husbands may use force to reprimand their wives because men should be in control. This is a statement about the appropriateness of IPV, not about seeking help following the experience of IPV. In this case, the item is retained based on its psychometric fit with other items, but this would also seem to merit more discussion. Why was this item selected for testing? Did the authors evaluate Cronbach's alpha when excluding this item? Note that the scale overall has an acceptable level of internal reliability, but arguably not an extremely high level.**

We draw this item as it was presented together along with items 2,3,4 in the existing IPV-Help Scale. Similarly to the authors of the IPV-Help Scale, we find this item, focused on tolerance of IPV, to be an important precursor to seeking help. If this attitude holds true, attitudes towards justifying seeking help for IPV are likely negative. We clarify that the four-item scale has an alpha of 0.71; when we drop the item "husbands may use force to reprimand their wives because men should be in control", the alpha drops to 0.67, suggesting this item increases internal consistency.

1.5 **Finally, it would be helpful to return again in the discussion to a comparison of this scale vis-a-vis the IPV-Help scale already evaluated in Nepal. The authors note that the scale in this paper is analyzed for youth, but is there a reason to believe this scale is particularly appropriate for youth, or particularly appropriate in Kenya? Do these scales have different or overlapping functions in the broader IPV measurement space?**

Thank you for this comment; we have taken a shortened version of the IPV-Help scale and tested it among youth, inclusive of men. Authors who tested IPV-Help among married women in Nepal recommended further testing among youth and men. We have tried to make this clear in the introduction as well as the discussion, where we highlight the contribution of testing this adapted scale among youth (344-348). We acknowledge that this is an innovative space with very few measurement tools to draw from, and little detail on how existing tools work among youth. Study leads in Kenya felt it was useful to test a form of the IPV-Help scale among youth; we therefore adapted from a different population to see how it fits. Further adaptation and testing are needed, as we note in the discussion (366-370).

Reviewer #2: The paper is well written and could provide valuable evidence to support response and prevention to intimate partner violence. The objectives, data sources, methods and results need more and clearer explanation for this work to make the contribution it seeks to.

2.1 The discussion on help-seeking is limited to the impact that individual attitudes and social norms have on behavior. Other barriers to help seeking from formal institutions, such as the availability, proximity, and accessibility of services, quality and satisfaction with services provided, the legal framework such as mandatory reporting, and financial resources to enable help seeking, would be important to mention even if not the primary focus of the research.
We clarify that in Nairobi, accessibility to quality services is relatively strong compared to other contexts, and the Kenyan government has highlighted GBV response within the essential minimum package of Universal Health Coverage (lines in clean manuscript; 375-377). Despite availability, help-seeking is still low, suggesting the importance of focusing on attitudes and norms or other potential barriers. We have added a statement to make clear we recognize our focus on help-seeking attitudes as part of a broader set of barriers to help-seeking in paragraph 3 of our discussion (371-373).

2.2 The data sources for this analysis are unclear. It would help to say more about the primary data source(s), including an assessment of quality and validity for the purpose of this research. The section on Patient and Public Involvement could be subsumed into that as it appears to relate to the original data collection rather than this secondary analysis. Some commentary on the possibility to do this analysis to other existing data sources, such as the Demographic and Health Survey conducted in Kenya in 2014, which collected data on attitudes, experiences of IPV and help seeking.
Thank you for pointing out that this is unclear. We clarify that this is a secondary analysis with data recently collected by our team; the measures included in the present analysis were not included in the initial study aims, which we have clarified in the study sample section (160-161). We have also added more context into our measures and their usefulness to the Patient and Public Involvement Section, where we highlight that attitudes on IPV help-seeking are not available in commonly used surveys such as the Demographic and Health Survey (267-270).

2.3 Clearer explanation is needed on how the IPV Response Scale is constructed. In particular, how the ‘IPV measure’ and ‘Help-seeking measure’ are being used. ‘IPV Response Scale’ does not seem to be an appropriate title for something based only on attitudes to social norms. Especially as ‘response’ is such an established concept amongst practitioners working on IPV-related interventions.
Thank you for this point. We agree that response does not capture our items appropriately; we have changed the name of the scale throughout to “IPV Help-seeking Attitudes Scale.” We have also adjusted the language so that “response” is used appropriately throughout the manuscript. We further addressed this concern throughout the manuscript by: 1) clarifying the content of the scale in the aims section (142); 2) more clearly highlighting the items considered for scale development in the measures section (177-190); and 3) explaining that while we did report prevalence of help-seeking itself among young women who experienced IPV, this sample was too small for running associations with the scale (346-348).

2.4 Given the focus is on IPV related help-seeking, it could be more impactful to focus this analysis solely on the women respondents, or more clearly explain the value of including the men. With the focus on youth some mention of controlling behaviors and other forms of IPV that are routinely measured would help to highlight that forms of violence are often overlapping and may change during the life course, such as escalate during pregnancy.
Thank you for this comment. We find our measures among men to be a key piece of our analysis, and an area that fills a gap in the existing literature. In our background/ aims section of the paper, we have justified the inclusion of young men more clearly (133-137). In the limitations section, we have also added a note that our IPV measure is limited (401-405).

2.5 The reason for, methods used and the findings from the subnational (subcounty) analysis could be explained in more detail.
The inclusion of the sub-county aggregate scales was intended to proxy the community norm, so that we could measure aggregate/ normative effects rather than only individual-level attitudes. We have outlined this more clearly in the methods (210-214). We additionally better highlighted the different findings between individual-level and community-level in the discussion (348-341).

2.6 Page 3, line 12-13 mentions that interpersonal violence is a leading cause of DALY among youth. Looking at the data source, this appears to be mainly firearm violence among males, and does not have any relationship with IPV. Including this statement could be misleading.
We agree this doesn't fit well with our overall focus and have removed the sentence.

2.7 Page 3, line 26: the language "Identifying" survivors suggests a compromise of their privacy and confidentiality. The reference being quoted here appears to use language drawn from an outdated version of the 'United States strategy to prevent and respond to GBV globally'. I suggest quoting the primary source and updating the language to align with the 2022 version. Alternatively, consider referring to UN or other international guidance on the pillars of response and prevention.
We have changed the text to reflect the 2022 global strategy with relevant citation.

2.8 Page 3, line 34: check reference to the year 2006 as is inconsistent with the publication date of reference 19.
Thank you; we have fixed this to 2005 in the text.

2.9 Page 4, line 31: the objectives could be elaborated to explain why this work is being undertaken and how it will contribute to the field.
We added a preface to the objectives to help transition from the gaps discussed to the objectives (139-140).

2.10 Page 4, line 54: In the description of the study sample, it would be important to elaborate on the partnership status being used in this study; the questions used to ascertain partnership status; and the percentage of the original and 2021 sample were ever partnered (and therefore the focus on the study).
Thank you for this inquiry. In Table 1, we present the percent partnered overall and by gender among the analytic sample collected in 2021. We included all youth, partnered or not, in the analysis of norms and attitudes; though IPV was only assessed among partnered young women. We have added: "*Questions on IPV were asked only among partnered young women*." (166-167) to clarify this restriction. We have also added the percent in a romantic relationship among the original 2019 sample to the study sample section (158).

2.11 Page 5, line 48: provide reasons for why the last six months was used as a reference period as opposed to the standard last 12 months.
We have clarified in the methods section that this is secondary data analysis using pre-existing cohort data. Since the survey round that we use was approximately 6 months after the previous survey round, this was the referent period due to time-sensitive study objectives related to the Covid pandemic.

2.12 Page 8, line 26: consider using "intimate partner" in place of "romantic partner" for consistency.
We have edited this wording for consistency.

2.13 Consider updating reference 2 to the 2018 WHO global estimates (Violence against women prevalence estimates, 2018: global, regional and national prevalence estimates for intimate partner violence against women and global and regional prevalence estimates for non-partner sexual violence against women. Geneva: World Health Organization; 2021. Licence: CC BY-NC-SA 3.0 IGO.)
Thank you. We have updated this reference.

Reviewer #3: The study provides comprehensive background with clear objectives and methodology. Approach to data analysis as well as applied factorization is well defined.

3.1 However, for the purpose of replicability of the study beyond specified geography of Nairobi, Kenya; inclusion of some additional points under methodology would strengthen the study e.g. process adopted to reduce the biases in case of self-reported data which is currently used in this study.
We clarify that this is a secondary data analysis of one cross-sectional round from a larger, ongoing cohort study in Nairobi, Kenya. Details on the study methodology for the ongoing cohort can be found in the cited report

(line in clean manuscript; 163).[1] The study began with a formative research phase in March 2019, which included focus group discussions with youth, youth organization leaders, and local stakeholders to solicit input on study feasibility and network subpopulations of interest. While the original round of the survey could be self-administered via a handheld tablet to maximize confidentiality, due to Covid-19, the 2021 survey round (which is the sample used in this analysis) was administered over the phone, in which the help-seeking attitude questions were first integrated in response to local needs. Privacy was ensured by enumerators and enumerators were highly trained on participant comfort and confidentiality during the data collection process.

3.2 In addition, description of recruitment or sampling of sub-group of YRDSS would be beneficial for easy replication of study. Overall, study objectives are relevant to the gender responsive system and it yields vital results.
The study utilized respondent-driven sampling (RDS), a chain-based recruitment method to identify high-risk, hard-to-reach individuals. RDS is premised on the assumption that peers are better able to locate and recruit other members of a hidden population than health facility or research staff. Eligible seeds and participants were unmarried adolescents aged 15-24 years who have resided in Nairobi for at least one year. Seeds were purposefully selected to serve as the initial contacts for recruiting from the target population through short interviews conducted by the local partner prior to study launch. Seeds were identified by local staff through personal networks and by the partner community-based organizations (CBOs) through their youth networks. Seed characteristics included sex, age, subcounty in Nairobi, level of schooling, and current school status (in-school or out-of-school). After selection and enrollment of initial seeds, recruitment of the target sample size was achieved through peer-to-peer coupon distribution. In the manuscript, we cite the study report that goes into detail on these procedures (163).[1]

Reviewer #4: Thank you for allowing me to review your manuscript. I am making some comments with the aim of improving this work. I have had difficulty reading your manuscript because I think there is a problem of structure.

4.1 Given that you present two different objectives, I think it would be more coherent to present the study in phases or stages. In the first phase present the development of the scale and in a second phase the analysis of the associations, in this way the work would gain in clarity. The research seems to be well conducted, but it is presented in a disorganized manner. In my comments I will therefore make this distinction between a first phase, which would be the development and evaluation of the properties of the scale, and in phase two, the analysis of the associations. Both phases should have a differentiated methodology and results.
Thank you for this suggestion; we have added sub-titles “Exploration of survey items”, “Scale refinement”, and “Scale associations” to both the methods and results section to make the organization clearer.

Stage 1 Create an IPV Response Scale that captures attitudes towards IPV and IPV help-seeking and examine its psychometric properties

4.2 The evaluation of psychometric properties of an instrument includes assessing content validation, construct, face validity, reliability (internal consistency and temporal stability, convergent-divergent validity among others. Indicate which psychometric properties have been finally examined and how.
We acknowledge limitations in our study due to this being an exploratory, secondary analysis study of the properties of two potentially applicable scales, previously tested elsewhere, rather than a scale development study. Unfortunately, our survey was not created specifically for this scale, and this was not a full scale-development process; we were adapting limited items from existing scales as a first step to exploring how help-seeking attitudes are related to IPV experience, to inform additional work in this area. We hope that substantial additions to our limitations section helps clarify (lines in clean manuscript; 383-391). Further, we have added clarification on the scope of our scale development process to our methods (224-228).

4.3 It seems that you have developed a scale from other existing scales. Indicate in a section which were the starting instruments and explain the process carried out: selected items, who made the selection of these and how the merging process was carried out. Cross-cultural and linguistic adaptation of the scales was made. In the end, do the selected items score differently? If this is the case, it has a decisive effect on the internal consistency and factor analysis.
We hope the additions to the limitations section discussed in the previous comment helps clarify the process behind the selection of items in the context of this secondary analysis. Item selection was informed by local priorities and Kenya-based colleagues working with youth in Nairobi (387-389). Respondents were given the

option to answer the survey questions in English or Swahili (165). Given we unfortunately do not have the full original scales in our data, we are unable to assess whether selected items score differently.

4.4 Explain and justify the estimated sample size.
As noted above, we have added clarity to our study sample section, limitations, and broader methods that this is secondary data analysis. We hope this clarifies the process by which the sample size was determined.

4.5a Indicate the approach used in the factor analysis (linear or nonlinear, Pearson's or polychoric correlation matrices) as well as the method of factor extraction.
Thank you for this note. We had previously used basic STATA factor analysis based on a matrix of Person's linear correlations. However, we investigated this further and found a more accurate approach is to run factor analysis after generating a polychoric correlation matrix given our variables are ordinal. Therefore, we have updated our factor analysis, observing slight changes to factor loadings in Table 3, but no changes to overall findings, and noted this approach within the methods section (224-226).

4.5b You can justify with references the system that you indicate of multiplying each survey item response by its loading based factorial load to create from there an adjusted scale.
We have reviewed our approach and after some consideration have decided to exclude the weighted scale from the analysis since it was adding limited value. Further, there are divergent views in the field as to how valuable this approach is; to save space we have removed output associated with the previous weighted scale given considerations about the contribution to the field.

4.6 Why didn't you calculate the omega coefficient instead of the cronbach coefficient. It is better, especially when working with factor loadings.
Thank you for this point. We have added the Omega reliability coefficient (227-228) and report within the results (305-306) and within Table 3; we find no substantial differences between the Alpha and Omega coefficient.

References

1. International Centre for Reproductive Health-Kenya (ICRHK) & PMA Agile. *Nairobi Youth Respondent-Driven Sampling Survey: Final Report. 2020. Performance Monitoring for Action Technical Report.* Baltimore, MD, USA: Bill & Melinda Gates Institute for Population and Reproductive Health, Johns Hopkins University Bloomberg School of Public Health;2020.

VERSION 2 - REVIEW

**Reviewer**	**1**
**Name**	**Leight, Jessica**
**Affiliation**	**International Food Policy Research Institute**
**Date**	**24-Sep-2024**
**COI**	

Thank you for the revised manuscript. It is thorough and I don't have any further comments.

**Reviewer**	**4**
**Name**	**Gonzalez-de la Torre, Hector**
**Affiliation**	**Universidad de Las Palmas de Gran Canaria, Enfermeria**

Date **25-Sep-2024**

COI

Dear Authors:

Thank you for allowing me to review the manuscript. I appreciate the effort you have made, as you have implemented most of the suggestions I made in the first revision. I am especially glad that you have changed the approach to factor analysis and adopted a more appropriate approach. On reflection, I would have liked a more formal process of development and validation of the scale-tool, although I understand that sometimes you start from data already collected and need to be flexible to adapt your research. I hope you will take this into account in future research on the topic. I think that the manuscript has gained in clarity with the contributions of the reviewers and the editor. For my part it can be published. Best regards
